# Evaluation of the Safety Profile of the ASFV Vaccine Candidate ASFV-G-ΔI177L

**DOI:** 10.3390/v14050896

**Published:** 2022-04-25

**Authors:** Xuan Hanh Tran, Le Thi Thu Phuong, Nguyen Quang Huy, Do Thanh Thuy, Van Dung Nguyen, Pham Hào Quang, Quách Võ Ngôn, Ayushi Rai, Cyril G. Gay, Douglas Paul Gladue, Manuel Victor Borca

**Affiliations:** 1 National Veterinary Joint Stock Company (NAVETCO), Ho Chi Minh City 70000, Vietnam; lephuong.navetco@gmail.com (L.T.T.P.); nguyenquanghuy166@gmail.com (N.Q.H.); dothuy81@gmail.com (D.T.T.); dungngvnavetco@gmail.com (N.V.D.); phamhaoquang@gmail.com (P.H.Q.); qngonnavetco@gmail.com (Q.V.N.); 2 Plum Island Animal Disease Center, Agricultural Research Service, U.S. Department of Agriculture, Greenport, NY 11944, USA; ayushi.rai@usda.gov; 3 Agricultural Research Service, U.S. Department of Agriculture, Beltsville, MD 20705, USA; cyril.gay@usda.gov

**Keywords:** ASFV, I177L, African swine fever, ASF, vaccine, swine, reversion to virulence

## Abstract

African swine fever (ASF) is the cause of a recent pandemic that is posing a threat to much of the world swine production. The etiological agent, ASF virus (ASFV), infects domestic and wild swine, producing a variety of clinical presentations depending on the virus strain and the genetic background of the pigs infected. No commercial vaccine is currently available, although recombinant live attenuated vaccine candidates have been shown to be efficacious. In addition to determining efficacy, it is paramount to evaluate the safety profile of a live attenuated vaccine. The presence of residual virulence and the possibility of reversion to virulence are two of the concerns that must be evaluated in the development of live attenuated vaccines. Here we evaluate the safety profile of an efficacious live attenuated vaccine candidate, ASFV-G-ΔI177L. Results from safety studies showed that ASFV-G-ΔI177L remains genetically stable and phenotypically attenuated during a five-passage reversion to virulence study in domestic swine. In addition, large-scale experiments to detect virus shedding and transmission confirmed that even under varying conditions, ASFV-G-ΔI177L is a safe live attenuated vaccine.

## 1. Introduction

Since the escape of African swine fever virus (ASFV) from Africa to the Republic of Georgia in 2007, this highly contagious disease has continued to spread uncontrollably from the Caucasus to Central Europe, Asia, and reaching the Caribbean island of Hispaniola in 2021 [1]. The main method of control to stop the spread of the virus is early detection and depopulation of pigs. To date, a safe and efficacious ASF vaccine has yet to have been successfully developed and commercialized to protect swine and stop the spread of ASFV. For low- and middle-income countries, the availability of a vaccine is paramount, as depopulating affected swine herds is not logistically or economically feasible. ASFV is a structurally complex virus with a large DNA genome encoding more than 150 genes [2,3,4]. Experimental live attenuated vaccine viruses, generated by deleting virus genes associated with virulence, have been shown to be effective [4,5,6,7,8,9,10,11,12,13,14]. One of these vaccines, ASFV-G-ΔI177L [7], induces effective protection against its parental virulent virus strain, Georgia 2007, as well as a virulent Vietnamese field strain isolated in 2019 [15]. This vaccine has been selected for further development and commercialization, which includes a thorough assessment of its safety characteristics. Here we present the results of safety studies conducted to assess the safety profile of ASFV-G-ΔI177L. Results showed that ASFV-G-ΔI177L inoculated in pigs 6–8 weeks of age is safe and lacks the ability to induce local and systemic disease. Moreover, although a low level of shedding from vaccinated animals could be detected for a limited number of days, the vaccine virus was shown to remain genetically stable and attenuated in back-passage reversion to virulence studies.

## 2. Materials and Methods

### 2.1. Cell Culture and Viruses

Peripheral blood mononuclear cells (PBMCs) were prepared from fresh defibrinated swine blood as previously described [15]. Briefly, ethylenediaminetetraacetic acid (EDTA)-treated swine blood was mixed with red blood cell (RBC) lysis buffer (155 mM NH_4_Cl, 10 mMKHCO_3_, 0.1 mM EDTA) at room temperature for 15 min and centrifuged at 2000 rpm for 10 min to remove the lysis buffer. The pellet was washed two times in medium or phosphate buffered saline (PBS). The cells were resuspended in media composed of RPMI 1640 Medium (Life Technologies, Carlsbad, CA, USA) with 20% L929 conditioned supernatant and 12.5% fetal bovine serum (Life Technologies). The cells were seeded at 150 × 10^6^ per T75 cm^2^ flask and incubated at 37 °C in a humidified 5% CO_2_ incubator for 3–4 days before infection. Virus titration was performed on primary swine macrophage cell cultures in 96-well plates as previously described [16]. All virus dilutions were performed using macrophage medium. The presence of virus was assessed by hemadsorption (HA), and virus titers were calculated by the Reed and Muench method [17]. ASFV-G-ΔI177L was developed as previously reported [7].

### 2.2. Quantitative Real Time PCR Protocol for the Detection of ASFV Genome

DNA extraction was performed using a Patho Gene-spin DNA/RNA Extraction Kit (Intron Biotechnology, Gyeonggi, Korea) for nasal swab samples or a QIAamp DNA Blood Mini Kit (Qiagen, Hilden, Germany) for blood samples. Detection of ASFV, using B646L (encoding for p72) as the target gene, was carried out using the VetMAX African Swine Fever Virus Detection Kit (Thermo Fisher Scientific, Waltham, MA, USA) as previously described [15]. Samples with Ct values < 45 were considered positive.

### 2.3. Next-Generation Sequencing (NGS) of ASFV Genomes

ASFV DNA was extracted from infected cells and quantified as described earlier. A full-length sequence of the virus genome was performed as described previously [6] using an Illumina NextSeq500 sequencer and by passage of the blood samples in primary swine macrophages one time to obtain enough ASFV genomic DNA for sequencing. Sequencing analysis was performed using CLC Genomics Workbench version 21.0.4, where sequencing reads were aligned to a reference genome. Basic variant detection was performed using a threshold of 90% with default parameters. A consensus genome was extracted using a low-coverage filter of 10 reads to determine the low-coverage areas in the sample.

### 2.4. Animal Experiments

Unless otherwise specified, all animal experiments were performed using crossbreed European Yorkshire and Landrace animals (produced at local farms). The pigs were transported to a holding pen and tested for the presence of ASF, CSF, PRRS, FMD, PCV2,3, mycoplasma antigens, and ASF antibody. Only pigs negative with these agents were used for experiments. Unless specifically denoted, all animal experiments were conducted inside the NAVETCO facility under controlled experimental conditions, following VICH guidelines for target animal safety studies: VICH GL 44 (Target Animal Safety), Biologicals, Ref. EMEA/CVMP/VICH/359665/2005 and VICH GL 41 (Target Animal Safety): Reversion to Virulence, Ref. EMEA/CVMP/VICH/1052/2004. The design of each of the experiments, including the age of the animals used, are described in Section 3. Unless otherwise specified, the doses used in the safety studies were the projected release dose for ASFV-G-ΔI177L production serials. All inoculations were performed intramuscularly (IM). Clinical signs (anorexia, depression, fever, purple skin discoloration, staggering gait, diarrhea, and cough) and changes in body temperature were recorded daily throughout the experiment. The presence of clinical signs associated with the disease was recorded as described previously [18].

### 2.5. Detection of Anti-ASFV Antibodies

ASFV antibody detection was performed using a commercial ELISA (Ingezim PPA, Ingenasa, Madrid, Spain) strictly following the manufacturer’s instructions. Values are presented as percent (%) of reactivity (positive when ≥50% and negative when ≤40%).

## 3. Results and Discussion

### 3.1. Evaluation of Virus Shedding in ASFV-G-ΔI177L Inoculated Pigs and Transmission to Naïve Pigs

The purpose of this study was to detect the shedding of vaccine virus secreted from pigs inoculated with ASFV-G-ΔI177L and to assess the transmission of virus to naïve pigs co-mingling with vaccinated pigs under laboratory conditions. All animals in this experiment were 7–8 weeks of age. A group of four animals (named I1–I4) were IM inoculated with 10^2.6^ HAD_50_/mL per animal. Immediately after inoculation, a group of five naïve animals (named C1–C5) was placed in contact with the inoculated animals. All animals were comingled during 28 days after inoculation and were monitored daily, looking for the appearance of clinical signs and rectal body temperature. The presence of virus in blood and nasal swabs in each of the animals of the inoculated and contact groups were tested by real-time PCR at 7, 11, 14, 21, and 28 days pi. The presence of ASFV-specific antibodies was tested in sera of all the animals at similar time points after inoculation using a commercial ELISA test.

No animals in the room, neither the inoculated nor the contacts, developed any clinical sign of ASF during the 28-day observational period.

The presence of the ASFV-G-ΔI177L genome was detected at low levels in the blood of all inoculated animals by day 7 pi (Table 1). The levels of virus genomic copies slightly increased in all animals by days 11, 14, and 21 pi, but by day 28 pi, all the animals became negative. The detection of the ASFV-G-ΔI177L genome in nasal swabs of the inoculated animals presented a more heterogeneous pattern. The presence of the virus was detected in pig I1 at days 11 and 28 pi, in pig I3 at 14 and 21 days pi, and in pig I4 at days 11 and 21 pi. Pig I2 remained negative during the experimental period. Therefore, although all inoculated animals became systemically infected after IM inoculation with ASFV-G-ΔI177L, detection of the virus in the nasal cavity was sporadic and inconsistent. Importantly, it was not possible to detect the presence of the ASFV-G-ΔI177L genome in any of the blood samples obtained from the contact comingled naïve pigs at any of the times evaluated. Two animals, pigs C2 and C4, presented the virus genome in nasal swabs, and only at 28 days pi. It is clear that although the genome of the vaccine virus could be detected at some of the tested times in all of the inoculated animals, none of the contact naïve pigs became systemically infected during the experiment.

The presence of ASFV-specific antibody response in sera of naïve contact pigs was also evaluated as evidence of potential replication of ASFV-G-ΔI177L. Importantly, sera samples obtained at 14, 21, 28, 35, 42, and 49 days pi of the naïve contact animals were all negative at all time points tested. This result supports the absence of systemic replication of ASFV-G-ΔI177L in the contact comingled animals, even though animals C2 and C4 showed the presence of the virus genome in nasal swabs at 28 days pi.

### 3.2. Virus Transmission under Field Conditions

An additional experiment to evaluate virus transmission from ASFV-G-ΔI177L-inoculated pigs to naïve animals was performed under field conditions. This experiment used animals at 10 weeks of age. In this study, a group of 50 animals was IM inoculated with ASFV-G-ΔI177L twice (with a three-week interval) with 10^2.6^ HAD_50_/mL per animal/dose. A group of 10 naïve animals was allowed to freely comingle with the vaccine-inoculated animals right after they received the first inoculation. An additional group of 20 naïve animals was included as a control and kept separately from the group of inoculated and contact animals. All animals were observed daily for 49 days pi to evaluate the appearance of clinical signs related to ASF. The presence of the virus in the blood in each of the contact and control animals was tested by real-time PCR at days 0 and 49 of the experiment. The presence of ASFV-specific antibodies was tested in sera of all the animals at similar time points after inoculation using a commercial ELISA test.

All 80 animals involved in the experiment remained clinically normal during the 49-day observational period.

All animals in the control group presented an absence of the ASFV-G-ΔI177L genome detected by PCR when tested both at day 0 and day 49 of the experiment (Table 2). As expected, all animals in the comingled contact group were negative by PCR when tested at day 0 pi, but three of them presented moderate levels of the ASFV-G-ΔI177L genome when tested at the end of the experiment (49 days pi). When evaluating the presence of ASFV-specific antibodies, as expected, all 80 animals were negative at day 0 pi, but 96% of the ASFV-G-ΔI177L-inoculated animals (24 out of 25 randomly selected animals out of the 50 initially inoculated) showed positive ELISA results at day 49 pi. In addition, whereas all control animals remained negative until the end of the experiment, 50% of the animals in the comingled contact group developed ASFV-specific antibodies. Therefore, ASFV-G-ΔI177L transmission under field conditions appears to confirm a certain level of transmission that is more efficient, since some contact animals actually present systemic virus replication and induction of an ASFV-specific immune response.

### 3.3. Overdose Test for ASFV-G-ΔI177L

In this study, we evaluated the presence of local and systemic disease in animals 7 to 8 weeks old inoculated with different doses of ASFV-G-ΔI177L. Considering 10^2.6^ HAD_50_ as a 1X dose of ASFV-G-ΔI177L per animal (as was determined to be the produced vaccine dosage), two different groups of animals were inoculated once with either a vaccine dose that was 10- (10X dose) or 5 (5X dose)-fold higher. Therefore, 14 animals (named 10XA-10XN) received 10^3.6^ HAD_50_, whereas six animals (named 5XA-5XF) were inoculated with 10^3.3^ HAD_50_ of ASFV-G-ΔI177L. An additional third group (12 animals, named 1XA-1XJ) received a 1X dose (10^2.6^ HAD_50_) of ASFV-G-ΔI177L twice, at 0 and 14 days pi. Animals in the three groups were IM inoculated and observed daily for 14 days after the last ASFV-G-ΔI177L inoculation, recording the appearance of clinical signs and rectal body temperature values.

**Figure 1:**
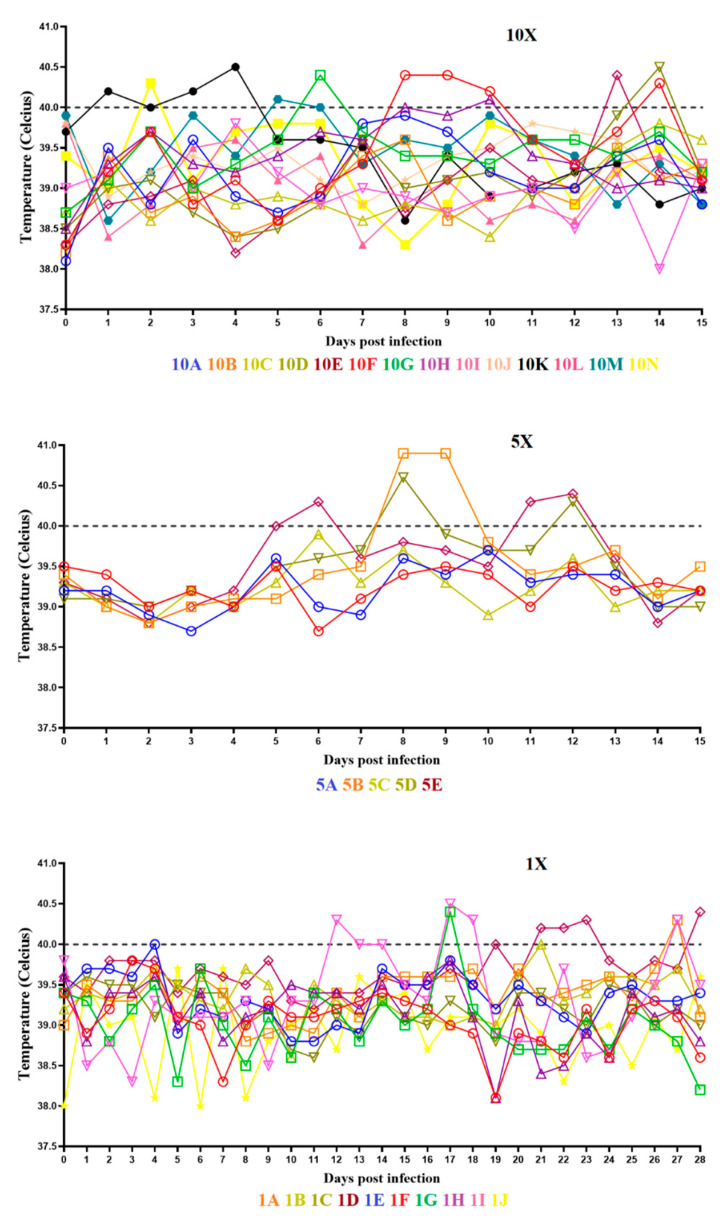
Evolution of body temperature in animals IM inoculated with either 10^3.6^ HAD_50_ (panel 10X), 10^3.3^ HAD_50_ (panel 5X), or 10^2.6^ HAD_50_ (panel 1X) of ASFV-G-∆I177L. Data are presented as individual values (expressed as °C) for each of the inoculated animals in each of the different groups.

Animals in the group inoculated with a 10X dose of ASFV-G-ΔI177L did not show the presence of any clinical signs associated with ASF, although some animals had occasional coughing for one day (animals 10XC on day 12 pi and 10XL on day 4 pi) or two days (animals 10XF on day 8–9 pi and 10XK on day 2–3 pi) or the presence of soft stools for one day (animal 10XK on day 15 pi) or for two days (animal 10XE on day 12–13 pi). The evolution of body temperature values in these animals remained normal except for very transient minor increases in temperature in some of the animals without being accompanied by the presence of any clinical sign of ASF (Figure 1, panel 10X).

Similarly, animals in the group inoculated with a 5X dose of ASFV-G-ΔI177L had no ASF-related clinical signs except during a two-day period of infrequent coughing in animal 5XB on day 8 and 9 pi, and animal 5XE presenting soft stools during days 11 and 12 pi. 

Animals receiving two 1X doses of ASFV-G-ΔI177L were observed for a total of 28 days after the first inoculation (14 days after the second inoculation). No important clinical signs related to ASF were observed in any of the animals observed. Only three animals showed either a transient presence of occasional coughing (animals 1XD and 1XI at days 21 and 2 pi, respectively) or the presence of soft tools in animal 1XB at day 20 pi. In addition, the values of body temperature were within the normal range with transient and minor increases in some of the animals, with an absence any clinical sign related to ASF (Figure 1, panel 1X).

An additional experiment was performed to further confirm the safety profile of ASFV-G-ΔI177L involving two different groups of animals. A group of 25 animals (7–8 weeks of age) of European origin (as described in Section 2) was IM inoculated with 10^2.6^ HAD_50_ per animal of ASFV-G-ΔI177L. A second group, composed of 22 animals (8 weeks of age) belonging to the Vietnamese breed Mong Cai (obtained from the Centre for Veterinary Research, NAVETCO), received IM in the same dose twice (at 0 and 14 days pi).

Results demonstrated that no animals, observed for 28 days pi, presented any clinical sign related to ASF, with the exception of a two-day period with soft tools in only two animals of the second group, on days 11 and 12 pi.

Therefore, in 79 animals inoculated with different doses of ASFV-G-ΔI177L, none of them presented pathognomonic clinical signs of ASF. The occasional minor clinical signs observed in these experiments (cough and soft stools) were always transitorily present in a small percentage of animals. Similarly, slight increases in body temperature peaks also momentarily indicated that ASFV-G-ΔI177L showed no significant local or general side effects when inoculated in young pigs.

### 3.4. Reversion to Virulence Studies

This study was performed to analyze whether the potential reversion to virulence of the attenuated ASFV-G-ΔI177L live attenuated vaccine could occur with a more virulent phenotype, and the genetic stability of the vaccine virus stock after serial back passage in different groups of swine. As such, the study included the sequential passage of the ASFV-G-ΔI177L master seed virus in 6–8-week-old pigs. The first four groups included three pigs each (named A, B, and C), whereas the fifth group contained five animals (named A, B, C, D, and E). The first group of animals was intramuscularly (IM) inoculated with 10^2.8^ HAD_50_ of ASFV-G-ΔI177L per animal. Groups 2 to 5 were IM inoculated with 2 mL per animal of a pool of blood samples obtained from the animals of the previous back passage at days 11 and 14 post inoculation (pi), which were previously determined in preliminary studies to be the highest peaks of viremia. Each blood pool used for inoculation was tested by PCR to confirm the absence of potential contaminant agents (CSFV, FMDV, PRRSV, and PVC2). In each of the passages, inoculated animals were observed daily for 28 days pi, with the appearance of clinical signs and rectal body temperatures being recorded. The presence of infectious virus in the blood was evaluated at days 7, 11, 14, 21, and 28 pi by titration of the samples in swine macrophage cultures.

**Figure 2:**
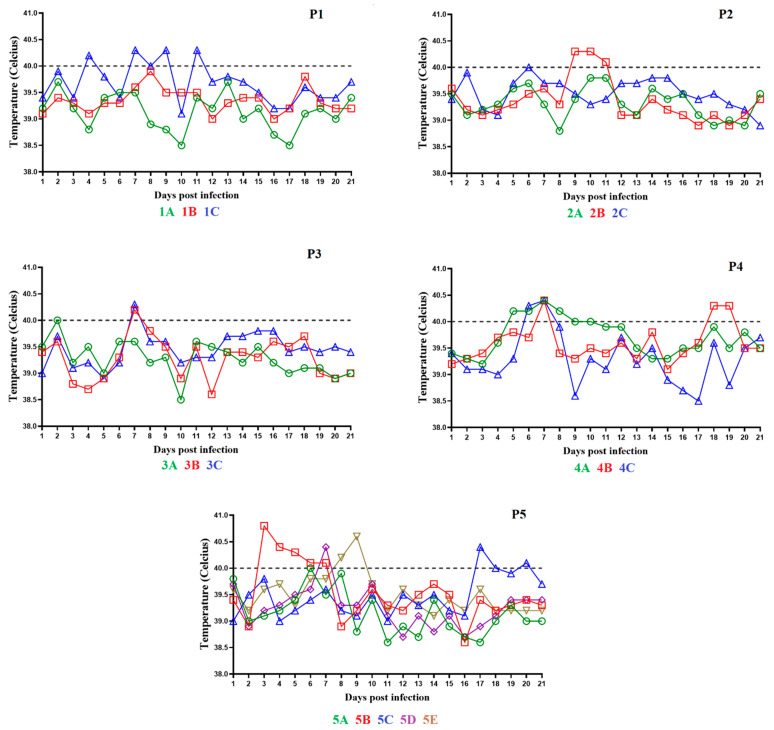
Evolution of body temperature in animals IM inoculated with ASFV-G-∆I177L during 5 consecutive passages (panels P1–P5, respectively). Data are presented as individual values (expressed as °C) for each of the inoculated animals in each of the different groups.

**Figure 3:**
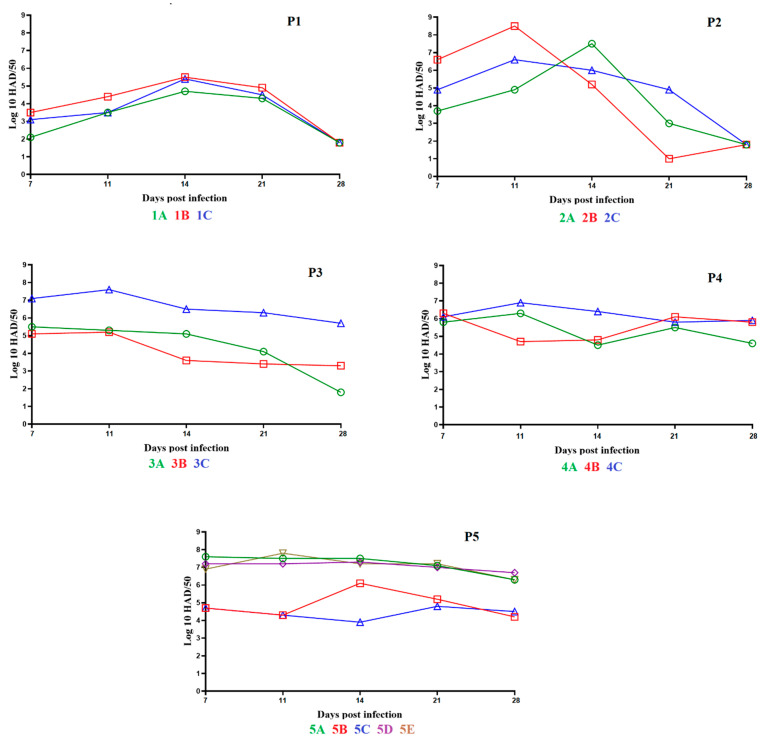
Evolution of viremia titers in animals IM inoculated with ASFV-G-∆I177L during 5 consecutive passages (panels P1 to P5, respectively). Data are presented as individual values of virus titers (expressed as HAD_50_/mL) for each of the inoculated animals in each of the different groups.

The animals in the first group remained clinically normal during the observational period with the exception of a three-day period (days 4 to 6 pi) when one of the pigs, 1B, presented soft stools. The animals showed normal body temperatures with the exception of some transient and minor increases in one of the animals, 1C, without the presence of any other clinical sign related to ASF (Figure 2, panel P1). Viremia titers in this group of animals showed low values (average of 10^2.9^ HAD_50_/mL) by day 7 pi, reaching middle values (average of 10^4.5^ HAD_50_/mL) by days 14–21 pi, which became undetectable by day 28 pi (Figure 3, panel P1).

In the second group, the animals were IM inoculated with 2 mL of pooled blood samples obtained from the first group of animals with a virus titer of 10^3.5^ HAD_50_/mL. After inoculation, the animals did not show clinical signs during the observational period with the exception of a three-day period (days 2 to 4 pi) when one of pigs, 2C, presented soft stools. All three animals showed normal body temperature with the exception of a transient mild increase in temperature in animal 2B, but that was not accompanied by the presence of any clinical signs of disease (Figure 2, panel P2). Viremia titers in this group of animals showed an average of 10^5.1^ HAD_50_/mL on day 7 pi, reaching average titers of 10^6.7^ and 10^6.2^ HAD_50_/mL on days 11 and 14 pi, respectively. Viremia titers lowered to an average of 10^3^ HAD_50_/mL on day 21 pi and, as in the first group, became undetectable by day 28 pi (Figure 3, panel P2).

The animals in the third group were IM inoculated with 2 mL of pooled blood samples from animals in the second group, with a titer of 10^6.3^ HAD_50_/mL. As in the previous groups, the animals did not show clinical signs during the observational period with the exception of a two-day period (days 13 and 14 pi) when pig 3C had soft stools. In addition, all three animals showed normal body temperature with the exception of one day of slight increase in body temperature for each of the animals (Figure 3, panel P3). Viremia titers in this group of animals showed an average of 10^5.9^ HAD^50^/mL on day 7 pi, reaching average titers of 10^6.7^, 10^5.1^, and 10^4.6^ HAD_50_/mL on days 11, 14, and 21 pi, respectively. Final viremia titers averaged 10^4.5^ HAD_50_/mL on day 28 pi (Figure 3, panel P3).

The fourth group of animals was IM inoculated with 2 mL of blood samples pooled from animals in the third group, with titer values of 10^5.7^ HAD_50_/mL. Animals under observation during the 28 days pi showed no clinical signs with the exception of pig 4A presenting soft stools during a two-day period (days 10 and 14 pi). In addition, two of the pigs, 4A and B, had an intermittent cough for two days (days 6 and 7 pi). All three animals showed normal body temperatures during the observational period with the exception of minor increases for five days for pig 4A and three days for pigs 4B and 4C (Figure 2, panel P4). Titers of viremia showed an average of 10^6.1^ HAD_50_/mL on day 7 pi and reaching average titers of 10^6^, 10^5.2^, 10^5.8^, and 10^5.1^ HAD_50_/mL on days 11, 14, 21, and 28 pi, respectively (Figure 3, panel P4).

In the fifth back passage, five animals were IM inoculated with 2 mL of pooled blood samples from pigs in the fourth back passage, with titer values of 10^6.6^ HAD_50_/mL. These five pigs showed no clinical signs during the observational period. Pig 5C had a transitory decreased intake of food for a four-day period (days 17 to 20 pi). As with the animals in back passage four, all animals showed normal body temperatures during the observational period, with the exception of short periods (five days for pig 5B, two days for pigs 5C and 5E, and one day for pigs 5A and 5D) during which the pigs had a minor increase in body temperature (Figure 2, panel P5). Titers of viremia had average titer values of between 10^6.1^ and 10^5.6^ HAD_50_/mL on day 7 to 28 days pi (Figure 3, panel P5).

All pigs in each of the five successive back passages reached the end of the observational period (28th day pi) showing no clinical sign attributable to ASF, including the absence of body temperatures over 40 °C (Figure 2). In addition, all pigs had normal food intake and were alert and responsive to external stimuli. Therefore, the attenuated phenotype of ASFV-G-ΔI177L remained stable throughout the five sequential back passages. The higher titers observed during back passages could have been due to the higher titer of the inoculum.

### 3.5. Assessment of Genomic Stability of ASFV-G-ΔI177L

The potential for genetic variations incurred with ASFV-G-ΔI177L during the five passages in the animals was assessed by comparing the full-length genome sequence of the stock virus used to inoculate the first group of pigs with the genome sequence of the vaccine virus isolated from the animals at the fifth passage (a pool of blood samples from all animals in group five). Variant detection was performed on sequencing reads for both viruses using a cut-off of 90%. The following minor differences were found in the virus obtained from animals in passage five related to the stock virus: A slight variation occurred in the poly C region of MGF 110-10-L-14L fusion protein where 91% of the population had one less C nucleotide. At position 86,225 there was a nucleotide change, G to A, that occurred in 99% of the sequencing reads, changing a single amino acid from a methionine to a threonine in the C257L open reading frame. The poly C region at the end of ORF MGF110-13lb was also shortened, with a reduction of 8 C nucleotides. The poly C and poly A changes that occur in a subpopulation of ASFV are not uncommon using our ASFV analysis pipeline for sequencing and analysis, and are either sequencing artifacts that occur often or represent the flexibility of these regions. In our cassette used to delete the I177L gene, there is a deletion of one A nucleotide in our p72 promoter used to drive the expression of our fluorescent reporter. Therefore, no major genomic differences were found between the stock virus and that obtained in passage five.

This report summarizes a group of experiments assessing different aspects of the safety profile of the vaccine candidate ASFV-G-ΔI177L. Results demonstrated that ASFV-G-ΔI177L does not present residual virulence, even when administered at doses 10 times higher than the minimal protective dose, not causing negative local or general clinical signs related to ASF. Only sporadic, transitory clinical signs (mild coughing, soft stools, or mild increase in body temperature) were identified during the experiments described in this report, with normal clinical conditions observed by the end of the study period. Importantly, the reversion to virulence experiment demonstrated the stability of the attenuated phenotype of the ASFV-G-ΔI177L vaccine through successive back passages in five groups of pigs. Young pigs, highly susceptible to infection with ASFV, were used in these experiments. Regarding shedding of ASFV-G-ΔI177L from vaccinated animals, we observed minimal or no transmission under controlled experimental conditions. No shedding was observed when the experiments were conducted under field conditions.

Based on the results of these safety studies, ASFV-G-ΔI177L was found to be a viable safe vaccine candidate with a stable attenuated phenotype, demonstrating the absence of clinical signs, reversion to virulence, or residual virulence associated with ASF.

## Figures and Tables

**Table 1:** Detection of virus genome by PCR in blood and nasal swabs of animals IM inoculated with ASFV-G-ΔI177L (I1–I4) and naïve contact animals (C1–C5). Results are expressed as negative (N) or Ct values.

Animal	Days Post Injection											
	0		7		11		14		21		28	
	Nasal	Blood	Nasal	Blood	Nasal	Blood	Nasal	Blood	Nasal	Blood	Nasal	Blood
I1	N	N	N	40.58	44.31	35.54	N	31.18	Nasal	33.01	32.78	N
I2	N	N	N	41.18	N	38.04	N	28.81	Nasal	32.19	N	N
I3	N	N	N	35.5	N	32.52	38.62	28.62	39.27	30.87	N	N
I4	N	N	N	37.23	40.7	35.58	N	28.9	38.23	32.24	N	N
C1	N	N	N	N	N	N	N	N	N	N	N	N
C2	N	N	N	N	N	N	N	N	N	N	36	N
C3	N	N	N	N	N	N	N	N	N	N	N	N
C4	N	N	N	N	N	N	N	N	N	N	36.96	N
C5	N	N	N	N	N	N	N	N	N	N	N	N

**Table 2:** Detection of virus genome by PCR (expressed as negative (N) or Ct values (ND: not determined)) and virus-specific antibodies (expressed as negative (N) or positive (P) values) in the blood of control animals or naïve contact animals comingling with IM inoculated with ASFV-G.

Groups (Number of Animals)	Real-Time PCR		ELISA	
	Day 0 pi	Day 49 pi	Day 0 pi	Day 49 pi
Control (20)	N *	N *	N *	N *
Contact (10)	N	37.29	N	P
	N	N	N	P
	N	36.9	N	P
	N	21.14	N	N
	N	N	N	P
	N	N	N	N
	N	N	N	N
	N	N	N	P
	N	N	N	N
	N	N	N	N
Vaccination (50)	ND	ND	N *	24/25 P

*: All animals in the group presented negative results.
